# Molecular and Medical Aspects of Psychedelics

**DOI:** 10.3390/ijms25010241

**Published:** 2023-12-23

**Authors:** Adam Wojtas, Krystyna Gołembiowska

**Affiliations:** Unit II, Department of Pharmacology, Maj Institute of Pharmacology Polish Academy of Sciences, 12 Smętna Street, 31-343 Kraków, Poland; wojtas@if-pan.krakow.pl

**Keywords:** psychedelics, neurotransmitter release, receptors, behavior

## Abstract

Psychedelics belong to the oldest psychoactive drugs. They arouse recent interest due to their therapeutic applications in the treatment of major depressive disorder, substance use disorder, end-of-life anxiety,= and anxiety symptoms, and obsessive–compulsive disorder. In this review, the current state of preclinical research on the mechanism of action, neurotoxicity, and behavioral impact of psychedelics is summarized. The effect of selective 5-HT2A receptor agonists, 25I- and 25B-NBOMe, after acute and repeated administration is characterized and compared with the effects of a less selective drug, psilocybin. The data show a significant effect of NBOMes on glutamatergic, dopaminergic, serotonergic, and cholinergic neurotransmission in the frontal cortex, striatum, and nucleus accumbens. The increases in extracellular levels of neurotransmitters were not dose-dependent, which most likely resulted from the stimulation of the 5-HT2A receptor and subsequent activation of the 5-HT2C receptors. This effect was also observed in the wet dog shake test and locomotor activity. Chronic administration of NBOMes elicited rapid development of tolerance, genotoxicity, and activation of microglia. Acute treatment with psilocybin affected monoaminergic and aminoacidic neurotransmitters in the frontal cortex, nucleus accumbens, and hippocampus but not in the amygdala. Psilocybin exhibited anxiolytic properties resulting from intensification of GABAergic neurotransmission. The data indicate that NBOMes as selective 5-HT2A agonists exert a significant effect on neurotransmission and behavior of rats while also inducing oxidative DNA damage. In contrast to NBOMes, the effects induced by psilocybin suggest a broader therapeutic index of this drug.

## 1. Introduction

Serotonergic hallucinogens might be the oldest psychoactive substances used by humanity. These substances, known for inducing profound changes in perception, mood, and cognitive processes, are often associated with therapeutic, religious, and recreational applications [1]. It is due to those properties that Humphrey Osmond coined the widely-used name “psychedelic”, originating from the Greek words psukhḗ, “mind”, and delos, “to reveal”. The modern era of psychedelic research began in the 19th century with the discovery of mescaline, the active ingredient of hallucinogenic Peyote cacti [2]. The next chapter opened in 1938 with synthesis and 5 years later the discovery of the psychoactive properties of lysergic acid diethylamide (LSD) [3]. Due to its extreme potency, LSD quickly became the most intensely researched psychedelic compound, with more than 1000 published articles by the end of the 1960s [4]. It was studied as a possible aid in psychotherapy, or otherwise explored as a potential treatment for substance abuse disorders, anxiety, and mood disorders [5]. Unfortunately, alongside medical use, recreational use of these substances swept across the globe, resulting in the passage of the “Controlled Substances Act “ in 1970 where psychedelics were classified as “drugs with no currently accepted medical use and a high potential for abuse”. These circumstances made it difficult to continue research concerning psychedelic drugs and nearly all studies (with only a few exceptions) came to an abrupt end, followed by several decades of hiatus in psychedelic research. However, in recent years, clinical trials have been conducted and several psychedelic drugs are indications for the treatment of major depressive disorder and treatment-resistant depression, substance use disorder, end-of-life anxiety, cancer-related depression and/or anxiety symptoms, and obsessive–compulsive disorder [6].

In this work, we review the current state of preclinical knowledge about the mechanism of action, receptor targets, and toxicity of psychedelics at the level of neurotransmission and behavioral response. Specifically, the NBOMe class and psilocybin are chosen in this review as these substances show different pharmacological properties and distinct medical potential.

## 2. Classification of Psychedelics

All psychedelic compounds can be divided by their structure into two main categories: phenylalkylamines, e.g., mescaline (3,4,5-trimethoxyphenethylamine) or DOI (2,5-dimethoxy-4-iodoamphetamine) [7] and indoleamines, e.g., DMT (*N*,*N*-dimethyltryptamine) or LSD. Both of them bear a resemblance to endogenous compounds—either phenethylamine or serotonin. While the former bind mainly to the 5-HT2 receptor family [8,9], the latter demonstrate an affinity for several types of receptors and nearly all 5-HT receptors [10].

The only hallucinogenic phenethylamine that exists in nature is mescaline, the active compound of *Peyote* and *Echinopsis* cacti [11], while the biggest group being the “2C” series act primarily as stimulants, though in higher doses they exert hallucinogenic effects [12]. Hallucinogenic amphetamines cannot be found in nature and have to be obtained through synthesis. Their best-known representatives are the 4-substituted-2,5-dimethoxyamphetamines, with the most important being 2,5-dimethoxy-4-iodoamphetamine (DOI), 2,5-dimethoxy-4-methylamphetamine (DOM), and 2,5-dimethoxy-4-bromoamphetamine (DOB) and (DOC). Due to their high affinity (nanomolar or even subnanomolar) for the 5-HT2A receptor, they have been used as radioligands to map the distribution of the aforementioned receptor in the brain [13]. A new class of serotonergic hallucinogens, *N*-(2-methoxybenzyl)-2,5-dimethoxy-4-substituted phenethylamines, in short NBOMes, became very popular as recreational drugs [4]. Many compounds belonging to this group have been synthesized, differing in substituents on the phenyl ring at position 4. The addition of the *N*-2-methoxybenzyl group significantly increased the affinity for the 5-HT2A serotonin receptor subtype (Table 1). Quite like phenethylamines, indoleamines can also be further differentiated into two subclasses: simple tryptamines like DMT or psilocin and ergolines (lysergamides), which are tryptamine derivatives with more rigid conformation; they primarily consist of LSD and its derivatives. Tryptamines are quite often encountered in nature. DMT can be found in *Ayahuasca*, a brew made from the leaves of *Psychotria viridis*; bufotenine (5-OH-DMT) is secreted by glands of *Bufo alvarius*, an American species of toad; and the most common forms include psilocybin and its primary active metabolite psilocin, the compounds originating from *Psilocybe* fungi, which can be found all around the globe [1,14]. Tryptamines show affinity for a large number of 5-HT receptors. This includes 5-HT1A with the affinity for this receptor being sometimes almost as high as for 5-HT1B, 5-HT1D, 5-HT1E, 5-HT2A, 5-HT2B, 5-HT2C, 5-HT5A, 5-HT6, and 5-HT7 (Table 1) [10]. In high concentrations, they also bind to α-adrenergic receptors, dopaminergic receptors, and serotonin transporters (SERT) [10]. Nevertheless, these compounds induce their hallucinogenic effects via activation of the 5-HT2A receptor. Finally, ergolines are derivatives of alkaloids secreted by the *ergot* fungi. Due to their tetracyclic structure, they happen to be more rigid than the usual “simple” tryptamines [15]. Their history is inseparably bound with their most famous representative—LSD. It is, up to this day, one of the most potent psychedelics [11,16]. Similarly to tryptamines, ergolines exhibit high affinity for 5-HT1A, 5-HT1B, 5-HT1D, 5-HT1E, 5-HT2A, 5-HT2B, 5-HT2C, 5-HT5A, 5-HT5B, 5-HT6, and 5-HT7 receptors (Table 1) [17]. What is more unique to them is that they also bind to the dopaminergic D1 and D2 receptors and adrenergic α1 and α2 receptors [17,18,19].

## 3. The Mechanism of Action of Psychedelics

### 3.1. 5-HT2A Receptor as a Primary Target for Psychedelics

Hallucinogens exert their psychoactive effects by acting as agonists for the cortical 5-HT2A receptor [13,22,23,24]. The 5-HT2A receptor belongs to the G-protein-coupled receptor (GPCR) family. It is coupled with the Gq/11 protein, and its activation leads to phosphoinositide hydrolysis resulting in the formation of diacylglycerol and inositol triphosphate, which leads to the mobilization of intracellular calcium and subsequent membrane depolarization [4]. Furthermore, the intensity of the psychedelic experience in humans is correlated with the occupancy of the 5-HT2A receptor, mainly in the prefrontal cortex (PFC) [25]. This activation of 5-HT2A receptors in the PFC launches a downstream cascade of changes in connectivity and alterations in blood flow across multiple regions of the brain, e.g., cingulate cortex, inferior parietal lobule, lateral temporal cortex, hippocampus (HP), thalamus, amygdala, and claustrum [26,27,28] involved in cognition, emotional processing, sensory perception, or even self-recognition and theory of mind processes [4,29]. What is interesting is that even though psychedelics activate 5-HT2A receptors on glutamatergic pyramidal neurons in the brain, this typically does not induce depolarization or generation of action potentials. Instead, there is an increase in glutamate release from depolarized neurons, resulting in recurrent activity [30]. Notably, Beique et al. [31] identified a subset of large neurons in the deep cortical layers that exhibited significant sensitivity to 5-HT. These neurons exhibited substantial membrane depolarizations, leading to spiking activity. Based on these findings, Martin and Nichols [32] isolated a specific subset of psychedelic-activated neurons from rat brains. They revealed that psychedelics directly stimulated only a minor proportion of 5-HT2A receptor-expressing excitatory neurons, especially in crucial brain areas, such as the PFC and claustrum. In particular, the psychedelic-responsive neurons exhibit elevated gene expression for the 5-HT2A receptor, which likely underlies their heightened sensitivity to psychedelics compared with other neurons. The authors postulate that this distinct neuron group acts as a “trigger population”. The activation of these neurons subsequently recruits other cell types including subpopulations of somatostatin or parvalbumin inhibitory GABAergic interneurons or astrocytes [32].

### 3.2. The Impact of 5-HT2A Receptor Activation on Behavior

The 5-HT2A receptor is a key player in inducing the psychedelic experience in humans and its activation is considered as a proxy for hallucinogenic effect in animal models [4]. The head twitch response/wet dog shake (HTR/WDS) test is based on this mechanism, exhibiting significant construct validity. The assay quantifies rapid, rhythmic head movements observed in rodents post-administration of psychedelic 5-HT2A receptor agonists [33]. While some false positives have been identified, such as fenfluramine, p-chloroamphetamine, and 5-hydroxytryptophan, the test predominantly exhibits specificity for 5-HT2A receptor agonists [15]. Furthermore, the HTR assay seems to be highly sensitive to 5-HT2A receptor agonists known to produce psychedelic effects in humans, proving its predictive validity. This is evidenced by the fact that the non-psychedelic 5-HT2A receptor agonist, namely lisuride, does not evoke the head twitch phenomenon [33]. Unfortunately, the face validity of the assay is poor, as humans do not exhibit head-twitching behavior after administration of psychoactive drugs.

## 4. NBOMes

*N*-2-methoxybenzyl substitution of 2-C compounds [11] drastically enhanced the binding to the 5-HT2A receptor, which resulted in the synthesis of a number of NBOMe agents differing in substituents on the phenyl ring at position 4. The examples of this group (shown in Table 2) are called in short 25I-NBOMe, 25B-NBOMe, and 25C-NBOMe and have in their molecule a substituent of halogens: iodine, bromine, or chlorine [34].

### 4.1. The Effects of NBOMes on Behavior Observed in Rodents

A study conducted on C57BL/6J mice demonstrated that 25I-NBOMe, administered at doses of 0.1, 0.3, and 1 mg/kg, was 14 times more potent than its “parent” compound, 2C-I, in inducing HTRs, and that this effect was fully reversible using volinserin, a selective 5-HT2A receptor antagonist [36]. Escalating doses of 25B-NBOMe (0.1–10 mg/kg) caused a hormetic response in the number of WDS episodes in Wistar Han rats [37], and this effect was replicated in C57BL/6J mice and was reversed by the 5-HT2A receptor antagonist, ketanserin [38].

A study by Gatch et al. [39] showed that 25I-, 25B-, and 25C-NBOMe dose-dependently reduced animal locomotor activity. When administered subcutaneously, 25I-NBOMe (0.03–3 mg/kg) produced a bell-shaped effect on C57BL/6J mouse activity [40]. In line with this finding, the researchers have also shown that low (0.001–1 mg/kg) and moderate (0.1–1 mg/kg) doses of 25I-NBOMe increase mobility, while a high dose (10 mg/kg) causes a sharp decline in the spontaneous motor activity of male ICR mice [41]. Similarly, 25B-NBOMe inhibited the motor activity of Wistar Han rats measured in an open field (0.3–3 mg/kg) [37]. This phenomenon is similar to the effects of another 5-HT2A receptor agonist, DOI, which modifies locomotor activity via a 5-HT2A/5HT2C-dependent mechanism. Low and moderate doses primarily activate the 5-HT2A receptors located on pyramidal neurons, leading to neuronal excitation and stimulation of locomotor behavior. As the plasma levels of the drug increase, it leads to the activation of the 5-HT2C receptors located on GABAergic interneurons, which results in the suppression of locomotor activity [42].

Some studies suggest the potentially addictive properties of the NBOMes. 25I-NBOMe (0.3 mg/kg) induced place preference in the conditioned place preference (CPP) test and increased vocalization frequency, in a similar way to methamphetamine, in male C57BL/6J mice. On the other hand, those effects were not replicated in self-administration experiments in Sprague Dawley rats (0.03 mg/kg/infusion) since this study reported weak addictive properties compared with place preference studies [43]. 25B-NBOMe at a dose of 1 mg/kg induced place preference in the CPP test in male C57BL/6J mice. Moreover, it produced statistically significant but weaker than those reported for methamphetamine responses in the self-administration procedure at doses of 0.03–0.3 mg/kg/infusion [38]. 25N-NBOMe increased place preference in male C57BL/6J mice at a dose of 3 mg/kg and also produced self-administration in Sprague Dawley rats (0.1 mg/kg/infusion) [44]. 25D-NBOMe (1 mg/kg) induced CPP and was readily self-administered (0.03 mg/kg/infusion) in rats [45].

The mechanism behind those behavioral symptoms seems to originate from a dopaminergic mechanism. 25B-NBOMe [37], 25I-NBOMe [46], 25N-NBOMe [44], and 25D-NBOMe [45] increased extracellular dopamine (DA) levels in the striatum (STR) and nucleus accumbens (NAC), the structures involved in the reward system. Furthermore, the administration of NBOMe compounds affected the protein levels of DA receptors, a hallmark of abuse potential. 25B-NBOMe elevated D1 receptor expression and decreased D2 receptor expression in the mouse NAC. 25N-NBOMe reduced the expression of D2 receptors both in NAC and dorsal STR. It also decreased the expression of DA transporter (DAT) in the NAC, while increasing its phosphorylation in the NAC and dorsal STR. Furthermore, the drug significantly reduced the expression of tyrosine hydroxylase (TH) in the NAC. 25D-NBOMe increased the expression of the dopamine receptor D1 while decreasing the expression of the D2 receptor, and downregulated DAT [45]. Taken together, these changes in dopaminergic activity, while not as potent as those induced by methamphetamine, may originate from a similar mechanism. The excessive DA levels in NAC would stimulate the D2 autoreceptors, which are involved in the regulation of DA release. Overstimulation of D2 receptors would then lead to their downregulation and reduction in the activity of DAT and TH [47].

### 4.2. The Effects of NBOMes Observed in Humans

NBOMes became available to drug users when they first appeared on the illicit drug market in 2010 [48,49]. Similarly to LSD, they are most often sold on blotter paper; sometimes, they are even sold as LSD. They are administered either nasally or orally (either by swallowing or sublingually) in small, sub-milligram doses [48]. The duration of action varies for each route of administration [36]. The effects of ingestion are usually an outcome of the activation of serotoninergic and adrenergic pathways and may include severe visual and auditory hallucinations, agitation, aggressiveness, long-lasting seizures, tachycardia, sweating, hypertension and hyperthermia, and psychotic/paranoid behavior [12,50,51,52]. Based on recent knowledge, the risk of NBOMes toxicity excludes these 5-HT2 receptor agonists from the treatment of a range of psychiatric disorders.

### 4.3. The Effects Exerted on Neurotransmitter Release by a Single Exposure to NBOMes

While there is scientific consensus that the primary mechanism behind the psychedelic effect of NBOMes is related to the increase in glutamatergic neurotransmission in the PFC [4,53,54,55], the effects exerted on other neurotransmitters are not so consistent and vary from drug to drug. The NBOMe class is reported to be extremely potent [55,56,57,58] in elevating the extracellular levels of DA, 5-HT, and glutamate after acute administration of 25I-NBOMe, and numerous WDS episodes were also seen.

The effect of administration of 25B-NBOMe is consistent with these findings as an increase in cortical extracellular glutamate levels was observed [37], and this effect was more robust than the one exerted by 25I-NBOMe [55]. Furthermore, 25B-NBOMe induced a similar number of WDS to 25I-NBOMe but with a three times smaller dose (0.3 mg/kg) [37]. Altogether, these findings confirm the stronger affinity and potency of 25B-NBOMe at the 5HT2A receptor, reported in vitro by Rickli et al. [20]. Like 25I-NBOMe [55], 25B-NBOMe significantly increased the extracellular 5-HT levels [37]. This phenomenon may be the reason for the serotonin syndrome reported in humans after ingestion of 25B-NBOMe [51]. Furthermore, it increased the volume of dopaminergic neurotransmission akin to 25I-NBOMe. Those changes observed in NAC and STR suggest that it might exhibit reinforcing properties [37].

**Figure 1:**
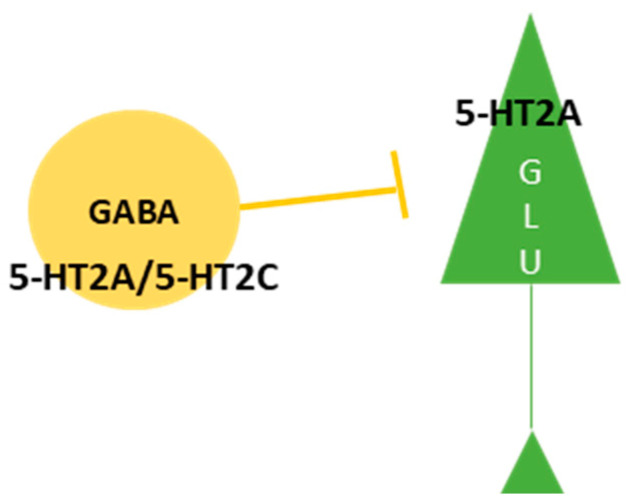
The location of the 5-HT2A and 5HT2C receptors in the cortical neurons.

The dose–response curve observed in microdialysis studies had an inverted “U” shape, most likely resulting from the activation of the different subtypes of 5-HT receptors. The progressive elevation of extracellular levels of neurotransmitters except acetylcholine (ACh), starting from the lowest dose (0.1 mg/kg) and reaching the plateau at 0.3 mg/kg, is a result of the activation of 5-HT2A receptors located on pyramidal glutamatergic cells [53,55,59,60]. As the plasma levels of the drug increase, subsequent activation of the 5-HT2C receptor located on cortical GABAergic interneurons occurs, leading to an increase in GABAergic neurotransmission and attenuation of the observed effect (Figure 1).

Both local [61] and systemic [62] administration of 5-HT2A/2C receptor agonists were reported to induce a dose-dependent increase in extracellular levels of GABA. This effect was further replicated when assessing hallucinogenic potency, with the WDS response curve which acquired a similar shape reaching the peak at the dose of 0.3 mg/kg [37], similar to 25I-NBOMe [55] and HTR in mice [38]. Furthermore, 25B-NBOMe reduced locomotor activity and induced anxiety in animals; these effects are also akin to those induced by 25I-NBOMe [46] and other psychedelics [42]. Surprisingly, while being more active at the 5-HT2A receptor than 25I-NBOME and inducing a greater increase in extracellular levels of neurotransmitters, 25B-NBOMe induced a very weak genotoxic effect evidenced by oxidative DNA damage in the nuclei from rat PFC and HP when compared with the control group [37] or when comparing it with the effect of 25I-NBOMe [63].

As reported by Quirion et al. [64], the 5-HT2A receptors are located on cortical cholinergic terminals, suggesting their role in the stimulation of ACh release. Nair and Gudelsky [65] reported that activation of the 5-HT2A receptor by DOI or mescaline stimulated ACh release in the PFC, and this effect was abolished by a 5-HT2A/B/C antagonist. In contrast to these results, the study by Wojtas et al. [37] observed a decrease, no changes, or increase in extracellular levels of cortical ACh after the administration of 25B-NBOMe, and the effect varied depending on the dose. These results are hard to explain due to a lack of data concerning the effects of psychedelics on cholinergic neurotransmission.

These results indicate that while being a powerful psychedelic capable of inducing significant enhancement of neurotransmission, which results in changes in animal behavior similar to those induced by other psychedelics, NBOMes might not be as toxic as other representatives of its class.

### 4.4. The Consequences of Repeated Exposure to NBOMes

As mentioned earlier, 25B-NBOMe elevates extracellular levels of DA in NAC and STR, which are elements of the reward system. Unlike other psychedelics, the NBOMes have been reported to exhibit rewarding properties, inducing CPP in mice and self-administration in rats [44,45]. Most importantly, a study by Custodio et al. [38] revealed that 25B-NBOMe also exhibited these properties in CPP and self-administration, and the effects were neutralized when using the D1 or D2 receptor antagonist, clearly suggesting the involvement of the dopaminergic system. Furthermore, chronic administration of 25B-NBOMe induced changes in the expression of the D1 and D2 receptors, namely upregulation of the former and downregulation of the latter, which is a common phenomenon induced by addictive compounds [38].

As stated before, frequent exposure to psychedelic drugs leads to rapid induction of tolerance [66,67], originating from the downregulation of the 5-HT2A receptor. This was also reported for 25I-NBOMe in rats [46] and 25B-NBOMe in mice [38]. Repeated administration of 25B-NBOMe completely suppressed its effect on cortical glutamatergic and dopaminergic neurotransmission and significantly reduced the effect it induced on extracellular serotonin levels [68]. This effect was more robust than the one elicited by 25I-NBOMe [46]. The influence of tolerance on the WDS response was similar to 25I-NBOMe, starting with a rapid decline in the number of WDS episodes on the second day of treatment. This effect can be explained by the downregulation of the 5-HT2A receptor, which diminishes the enhancement of glutamatergic neurotransmission in the PFC, an essential event needed for HTR/WDS to occur [4]. The observed decrease in locomotor activity after chronic exposure to 25B-NBOMe in the open field test may also be a result of tolerance. With the 5-HT2A receptor downregulation, the 5-HT2C receptor activation plays a greater role in modulating animal behavior, thereby leading to suppression of locomotor activity, as suggested by Halberstadt et al. [42].

The potency of 25B-NBOMe-induced increases in extracellular levels of the examined neurotransmitters was also reduced in NAC and STR. It is important to notice that in the NAC, the observed tolerance was much smaller [68]. Importantly, after a 7-day treatment, the compound still increased extracellular levels of dopamine, which corresponds with its effect on DA receptors in NAC reported in mice [38] and increased Δ-fosB expression. Taken together, these findings suggest that 25B-NBOMe might exhibit addictive properties like other representatives of its family. Nevertheless, it is worth noticing that these effects, while significant, are much smaller than the addictive properties of other classes of drugs of abuse, e.g., psychostimulants [38].

While acute administration of 25B-NBOMe resulted only in a minor genotoxic effect [37], it produced significant DNA damage in both PFC and HP after chronic treatment [68], in a similar way to 25I-NBOMe [64]. Repeated administration of 25B-NBOMe increased basal levels of glutamate, DA, and 5-HT, suggesting the appearance of maladaptive changes in neurotransmission. These increases might lead to genotoxicity directly when elevated glutamate levels overstimulate ionotropic glutamatergic receptors, resulting in DNA damage [69], and indirectly, when an excess of monoamines leads to disturbances in their metabolism, thus increasing the production of free radicals [69,70]. Moreover, phenethylamines may directly produce oxidative stress, which was shown by Xu et al. [71] in in vitro studies. Nevertheless, the observed genotoxic effect did not translate into permanent loss of brain tissue, measured as the volume of cortical and hippocampal regions [68]. Alongside the previous study, these results suggest that although it induces some DNA damage, the toxicity of 25-NBOMe cannot be explained by this phenomenon and is most likely a result of serotonin syndrome [72].

## 5. Psilocybin

### 5.1. History of Psilocybin

Psilocybin-containing mushrooms belong to a diverse group within the *Basidiomycota* fungi, comprising over 200 species across genera, such as *Psilocybe*, *Gymnopilus*, and *Panaeolus*, with the most prevalent genus being *Psilocybe*, with about 144 species [73]. Psychedelic mushrooms have been consumed and revered as religious objects since prehistoric times by many cultures, with evidence of such practices found in Europe, Africa, and most notably, Mesoamerica [74,75,76].

The modern history of psilocybin starts with Albert Hofmann, the chemist who discovered the psychedelic properties of LSD. In 1957, he received a sample of *Psilocybe Mexicana* mushrooms from which he extracted two crystalline compounds. Through self-experimentation, he confirmed their psychoactive effects, and by 1958, he had identified these compounds as psilocybin and psilocin, which he synthesized in 1958 [77].

### 5.2. Pharmacology of Psilocybin

After oral intake, psilocybin is quickly dephosphorylated in the stomach or by alkaline phosphatase in the intestine, kidney, and potentially blood, forming psilocin, which easily penetrates the blood–brain barrier [78,79,80]. Studies in rodents confirm the almost complete conversion of psilocybin to psilocin before entering the bloodstream [81]. Both psilocybin and psilocin have similar psychotropic effects on humans at equimolar concentrations. Thus, psilocybin acts as a prodrug, with psilocin being the active agent responsible for its in vivo effects [82]. Moreover, inhibition of alkaline phosphatase completely suppresses the psychoactive effects of psilocybin administration [83], further supporting the hypothesis that psilocin is the primary psychoactive agent in hallucinogenic mushrooms [84].

As psilocin is structurally akin to the neurotransmitter serotonin, it undergoes a similar metabolism. It is further degraded in the liver by monoamine oxidase (MAO) or aldehyde dehydrogenase, leading to various intermediates and end products [85,86]. This is why MAO inhibitors are sometimes taken by users in an attempt to intensify psilocin’s hallucinogenic effects. Furthermore, psilocin itself may competitively inhibit MAO, which may lead to elevated brain 5-HT levels and a decrease in 5-hydroxyindoleacetic acid (5-HIAA) levels [87].

Like all psychedelics, psilocin has a strong affinity for 5-HT receptors in the brain, primarily acting as an agonist at the 5-HT2A, 5-HT2C, and 5-HT1A receptors. The psychedelic effects of psilocin are largely nullified by ketanserin, a 5-HT2A receptor antagonist, indicating the central role of the 5-HT2A receptor in its subjective effects [88,89,90]. The role of 5-HT1A receptors in the psychoactive effects of psilocin is yet to be examined. Furthermore, psilocin interacts with other serotonin receptors, including 5-HT1B, 5-HT1D, 5-HT2B, 5-HT5A, 5-HT6, and 5-HT7 [84].

The subjective and behavioral effects of psilocybin are influenced by interactions with non-serotonergic receptors. Research suggests that psilocybin indirectly elevates DA levels in the striatum, which is associated with depersonalization and euphoria [91]. Nonetheless, the dopaminergic system plays only a partial role in the psychological effects of psilocybin, as haloperidol, a nonselective DA receptor antagonist, reduces only about 30% of these symptoms [90]. Contrary to LSD, which binds to D2 receptors, psilocybin exhibits no affinity for dopamine D2 receptors [92,93]. While there is evidence suggesting that classical psychedelics can elevate dopaminergic transmission in human striatal regions, they do not significantly activate the NAC in positron emission tomography (PET) imaging studies. This observation aligns with the absence of data linking classical psychedelics to substance use disorder [90,91,94,95].

### 5.3. The Effects of Psilocybin Observed in Animal Studies

Both psilocybin and psilocin have been observed to evoke HTR and WDS in mice and rats, respectively [96,97], and the effect was absent in 5-HT2A receptor knockout mice [15]. Psilocybin fully substituted for psilocin, DOM, and LSD [98], and these effects were abolished by coadministration of volinserin, further confirming that the primary effects of psilocybin in HTR are mediated via the 5-HT2A receptor [98]. Psilocin dose-dependently (1.25–5.0 mg/kg) reduced locomotor activity in rats [10] and mice [98]. What is interesting is that the treatment with the selective 5-HT1A receptor antagonist, WAY-100635, completely reversed the effects of psilocin on the locomotor activity of mice. Furthermore, deletion of the 5-HT2A receptor gene did not affect the response induced by psilocin [42]. These findings suggest that the effect of psilocybin/psilocin on locomotor behavior may be primarily mediated via the 5-HT1A receptor and not the 5-HT2A receptor.

To date, there are no animal studies suggesting that psilocybin exhibits addictive properties. Fantegrossi et al. [99] reported no significant differences between psilocybin and saline in self-administration in rhesus monkeys. A microdialysis study by Sakashita et al. [100] demonstrated that psilocin elevated extracellular levels of DA in NAC in a slight but significant way but did not affect them in the ventral tegmental area (VTA), which suggests rather low reinforcing properties. Furthermore, psilocin inhibited methamphetamine-induced conditioned place preference formation during the acquisition phase [101]. It also reduced intracranial self-stimulation [102], and this effect was only partially reduced by the administration of volinserin, suggesting that other receptors, besides the 5-HT2A receptor, may also contribute to this effect [103]. These experiments are consistent with the general view that classic psychedelics lack reinforcing properties [4] and suggest a possible use of psilocybin in the treatment of addictive disorders.

Acute administration of psilocin has been reported to either increase anxiety, measured as the center avoidance in mice [97], or exert no effect in this paradigm [104]. The anxiogenic effect was also reported in rats, but it diminished with repeated exposure to the experimental arena, suggesting that the anxiety is a result of drug-enhanced neophobia [15]. A recent study by Hibicke et al. [105] demonstrated the anxiolytic properties of psilocybin if the animals were repeatedly exposed to the novel environment. Anxiety was not observed in rats 24 h after psilocybin administration either in the open field or light/dark box (L/D) paradigm [62]. Furthermore, psilocybin/psilocin facilitated fear extinction in rats [106,107].

Psilocybin tested acutely in Flinders Sensitive Line rats [108] and 24 h after administration in naive controls [62] did not exert an antidepressant effect in the forced swim test (FST) but demonstrated a significant antidepressant effect 5 weeks after its administration [105]. Furthermore, it attenuated learned helplessness in mice 24 h after the drug administration [109] and reversed anhedonia in chronically stressed mice [110], while not affecting the immobility time measured in the FST. These findings support the thesis that (a) psilocybin/psilocin may exhibit antidepressant properties and (b) FST might not be suitable for evaluating the antidepressant qualities of psychedelics.

### 5.4. The Effects of Psilocybin Observed in Humans

Psilocybin has demonstrated minimal toxicity in animals, with an LD50 in rodents being 2000 to 3000 times the standard human dose on a mg/kg basis [84]. When assessing the acute toxicity, safety, and addictive potential of various psychoactive substances, psilocybin is consistently ranked at the lower harm end while, in comparison, opioids, notably heroin, are at the higher harm end [111,112,113]. Physiologically, psilocybin poses minimal risk to humans, showing no association with major organ damage, carcinogenicity, teratogenicity, enduring neuropsychological deficits, or overdose fatalities [76].

Oral psilocybin doses range from 0.045–0.429 mg/kg, with the psychedelic effect observed with oral doses > 15 mg [114] or plasma psilocin levels of 4–6 ng/mL [80]. Doses exceeding 25 mg orally are considered high but not dangerous [76]. After oral intake, onset of action is 20–40 min, peaking at 60–90 min with effects lasting 4–6 h, and complete cessation by 24 h [90,114].

Psilocybin administration results in dose-dependent effects: low doses produce drowsiness and amplify pre-existing moods [114]; medium doses instigate a controllable altered state of consciousness [82]; and high doses generate intense psychedelic experiences, characterized by altered perception, dream-like states, illusions, hallucinations, synesthesia, and alterations in the perception of time and space [95,114,115].

According to the Altered States of Consciousness scale (ASC) scale [116], when juxtaposed with ketamine, psilocybin exhibits pronounced visual hallucinatory effects, whereas ketamine more markedly disrupts physical integrity [93,117,118]. Pharmacological interventions have provided insights into the mechanism of action of psilocybin. Both ketanserin (5-HT2A/C antagonist) and risperidone (mixed 5-HT2A/C and D2 antagonist) have been observed to normalize psilocybin-induced alterations. Conversely, haloperidol, a D2 receptor antagonist, only modulates euphoria, derealization, and depersonalization but fails to influence visual hallucinations [90].

### 5.5. The Effects of Psilocybin as a Fast-Acting Antidepressant

**Figure 2:**
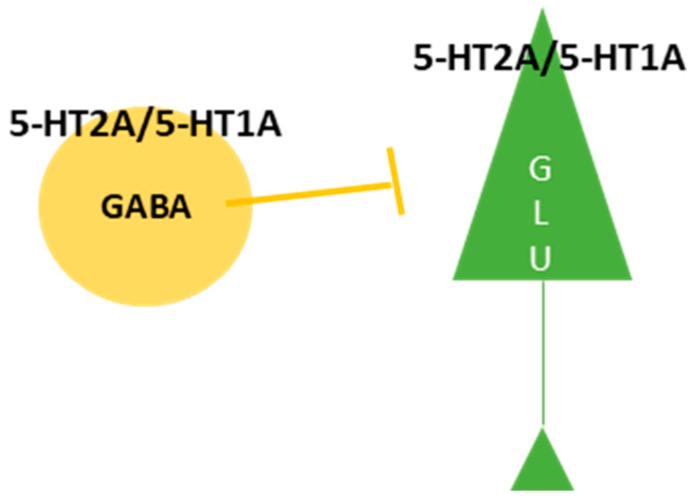
“Bipartite” model according to which 5-HT1A and 5-HT2A receptors play a crucial role in psilocybin-induced alterations in brain activity [129].

While possessing distinct pharmacological targets, namely, the 5-HT2A receptor for psychedelics and the NMDA receptor for ketamine, those drugs exhibit some overlapping properties. Either stimulation of cortical 5-HT2A receptors located on pyramidal cells by psychedelics or inhibition of cortical GABAergic interneurons by ketamine is supposed to result in a “glutamate surge”, which is a sudden elevation of cortical glutamate levels [119,120]. Subsequently, this phenomenon leads to an increase in neuroplasticity, producing an antidepressant effect [121,122]. The molecular mechanism underlying the neuroplastic effects of psychedelics involves 5-HT2A receptor signaling initiated by increased Ca^2+^ influx in the dendritic compartment that drives the release of neurotrophic factors (e.g., brain-derived neurotrophic factor, BDNF), which act on tropomyosin receptor kinase B (TrkB) receptors to stimulate mammalian target of rapamycin (mTOR) signaling crucial to synapse formation [109,119,123]. Overall, in this model alterations in glutamate release contribute to neuroplasticity. However, in addition to being agonists of the 5-HT2A receptor, psychedelics are also agonists of the 5-HT2C and 5-HT1A receptors [4]. 5-HT1A and 5-HT2A receptors are highly expressed and often co-expressed in PFC pyramidal neurons and GABAergic interneurons. Almost 60% of cells in the PFC contain mRNA for 5-HT1A and 5-HT2A receptors, with co-localization of up to 80% [124,125,126]. Thus, the cellular response is determined by the summation of 5-HT1A receptor-induced inhibition and 5-HT2A receptor-induced excitation. The 5-HT1A receptor signaling at GABAergic interneurons may inhibit this inhibitory control of pyramidal cells leading to their disinhibition. In this way, psilocybin modulates local and downstream effects. Psilocybin could also activate pyramidal neurons via 5-HT2C receptors expressed in layer V neurons of the PFC [127]. However, the depolarizing action of 5-HT in layer V pyramidal neurons of the PFC does not seem to depend on 5-HT2C receptor activation since it was not blocked by the selective antagonist SB 242084 [128]. According to the “bipartite” model proposed by Carhart-Harris and Nutt [129], 5-HT1A and 5-HT2A receptors play a crucial role in psilocybin-induced alterations in brain activity (Figure 2).

On the global level, the administration of psychedelics [130,131] reduces the activity of the default mode network (DMN), which is hyperactivated in depressive disorders. As stated by Vollenweider and Kometer [93], this translates into partially overlapping subjective experiences produced by psilocybin.

### 5.6. The Effect of Psilocybin Exerted on Cortical and Thalamic Neurotransmitters, Receptors, and Behavior

As reported in other works regarding psychedelics [37,53,55], psilocybin elevated the extracellular glutamate levels in the PFC, providing further evidence for the “glutamate surge” hypothesis [82]. Furthermore, it elevated cortical levels of DA only at a smaller dose, with the high dose being ineffective. This finding is in accordance with the work of Sakashita et al. [100], who reported a slight but significant decrease in cortical DA after administration of a similar dose (10 mg/kg) of psilocin. The authors explain this fact by the activation of 5-HT2C receptors, which are reported to decrease DA activity in the PFC [132]. Psilocybin dose-dependently raised the extracellular levels of 5-HT in the PFC [62], which was observed previously for psilocin by Sakashita et al. [100]. This effect might be mediated via the 5-HT2A receptor, as local administration of 5-HT2A agonist (DOI) into the PFC elevated extracellular levels of cortical 5-HT and this effect was neutralized with 5-HT2A receptor antagonist volinserin [133]. Furthermore, psilocybin dose-dependently increased GABA in the PFC [62]. What is important to note is that this also happens in humans after the administration of psilocybin, as stated by Mason et al. [134]. This could be explained by either the activation of the 5-HT2C [124] or 5-HT2A [4,135] receptor. Both are located on GABAergic interneurons in the PFC, and psilocin exhibits similar affinities for them [10].

**Figure 3:**
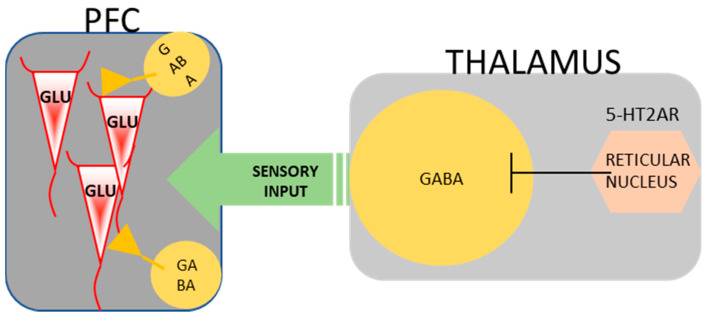
Filtering of sensory input into the cortex by the thalamus via 5-HT2A receptors [118].

As reported by Rodriguez et al. [136], 5-HT2A receptors exhibit high expression in the reticular nucleus (RN) of the thalamus, which is composed mainly of GABAergic interneurons. Vollenweider and Geyer [118] proposed that RN was responsible for filtering the thalamic input into the cortex and that psychedelics enhanced its GABAergic activity, disrupting the negative feedback. The observations of Wojtas et al. [62] seem to support this hypothesis, as a dose-dependent increase in GABAergic neurotransmission was reported while any significant effect on glutamatergic neurotransmission was not observed (Figure 3).

Due to the release of glutamate induced by the administration of psilocybin, the expression of GluN2A and GluN2B subunits of the NMDA receptor and GluA1 and GluA2 subunits of the AMPA receptor in the PFC 24 h after administration of psilocybin was examined. No significant effect was observed, except for the increase in expression of the GluN2A subunit when using the higher (10 mg/kg) dose of psilocybin [62]. These results are hard to explain, and perhaps it would be useful to replicate this experiment with different time points to evaluate if changes might happen earlier/later after the drug was administered.

While it is generally acclaimed that classical psychedelics are physiologically safe to use, a significant increase in glutamatergic activity in the PFC after high doses of psilocybin was observed, which may result in genotoxicity. To examine this, the oxidative DNA damage was assessed with the Comet Assay [62]. The 10 mg/kg dose of psilocybin significantly harmed the DNA in both the PFC and HP, suggesting caution when using high doses of this drug.

To investigate the long-lasting effect of psilocybin administration on rats’ behavior, behavioral tests were conducted 24 h after drug administration to ensure that the injected drug would leave the system. No effect on the locomotor activity was observed [62] when measured in the open field test and only a slight drop in the distance traveled was seen in the L/D box test after administration of 2 mg/kg of psilocybin. This suggests that after 24 h, the drug is washed out of the body and nearly no long-lasting effects are present, supporting the hypothesis about its safety. Since it did not produce an antidepressant effect in the forced swim test (FST), it is worth noticing that this assay may not be suitable for testing fast-acting antidepressant drugs [108].

### 5.7. The Limbic Response to Psilocybin

Alongside the PFC, the limbic system also exhibits maladaptive changes in depressive disorders [137]. Both the HP and amygdala undergo hypotrophy during depression, while NAC exhibits dysfunctional hypertrophy [137,138,139]. Recent studies have reported that the administration of ketamine is able to reverse those changes in all aforementioned structures [139,140], and these effects are correlated with antidepressant effect. The data concerning the effect of psilocybin on the limbic system and limbic-related behavior are scarce.

As reported by Sakashita et al. [100] and Wojtas et al. [141], psilocin dose-dependently increased extracellular DA levels in the NAC. This effect can be explained by the stimulation of 5-HT2A receptors, as local administration of 5-HT2A agonists enhances DA release in the NAC [142,143], while administration of 5-HT2A antagonists leads to inhibition of DA release in the NAC [143]. Interestingly, while Sakashita and colleagues [100] observed a slight but significant and dose-independent drop in the extracellular level of 5-HT in the NAC after administration of psilocin, Wojtas et al. [141] reported a dose-dependent increase. This effect might be explained by the stimulation of pyramidal cells, which send their projections to the raphe nuclei, and subsequent stimulation of serotonergic neurons projecting to the NAC [144].

Intriguingly, psilocybin decreased accumbal glutamate levels [141]. Previous studies by Wojtas et al. [37] with a more selective 5-HT2A agonist, 25B-NBOMe, reported increases in glutamatergic activity in the NAC. This suggests the involvement of other receptor subtypes, as psilocin is a more promiscuous drug, but the 5-HT1A receptors located on glutamatergic cells projecting into the NAC are the most probable target [145]. The observed increase in the extracellular level of GABA might result from the stimulation of 5-HT2A receptors located on GABAergic interneurons [146].

The opposing effect of lower and higher doses of psilocybin on extracellular levels of glutamate in the HP is hard to explain; the dose-dependent increase in GABA excludes the involvement of GABAergic interneurons [141]. Furthermore, psilocybin increased extracellular levels of ACh in the HP, with the lower dose being more potent [141]. This might be a result of subsequent activation of the 5-HT2A receptor, which stimulates ACh release [65], and then the 5-HT1B receptor, which inhibits ACh release in the rat HP [147]. Perhaps the inhibition of glutamatergic activity by the lower dose of psilocybin results from the elevated levels of ACh acting at M4 muscarinic receptors located on hippocampal pyramidal cells [148].

Psilocybin did not affect extracellular levels of glutamate and GABA in the amygdala [141]. 5-HT2A receptors are highly expressed on pyramidal cells and both parvalbumin and somatostatin GABAergic interneurons in the amygdala, which suggests that the effect of their activation may be mutually suppressive [149].

Despite the fact that psilocin is a 5-HT2A receptor agonist and it elevated the extracellular levels of 5-HT in the NAC, it did not affect the expression of the 5-HT2A receptor. This may be due to its low density in the NAC [150]. Changes were observed for the D2 receptor when using a higher dose of psilocybin [141]. This may be a result of an interplay between dopaminergic and serotonergic neurotransmission, as the administration of haloperidol attenuates the psychotomimetic effects induced by psilocybin administration [82].

Both 5-HT2A and 5-HT1A receptors show high-density expression in the HP [149,151]. The observed decrease in 5-HT1A receptor density might be a result of their stimulation either by psilocybin or the release of serotonin, but there is a lack of data regarding the latter [141]. The decrease in 5-HT2A receptor expression induced by the lower dose of psilocybin [141] may be a result of rapid downregulation, while the increase caused by the higher dose might be a result of an increase in synapto- and neurogenesis [123].

Psilocybin exhibited a dose-dependent anxiolytic effect measured as a decrease in center avoidance in the open field test both 1 and 24 h after the drug administration, and this effect might result from intensification of GABAergic neurotransmission observed in limbic structures [141].

### 5.8. The Therapeutic Potential of Psychedelics

Besides being a non-toxic and well-tolerated drug, over the last decade, psilocybin has been thoroughly examined as a potential fast-acting antidepressant drug. Carhart-Harris et al. [152] reported that acute administration of psilocybin produced significant effects in 67% of patients with treatment-resistant depression in the first week after the treatment, with 47% of treated individuals staying in remission for 3-month and 6-month periods [153]. A single dose of psilocybin was associated with a reduction in depressive symptoms without serious adverse events in a randomized clinical study of Raison et al. [154]. A study by Gukasyan et al. [155] reported long-lasting antidepressant effects of psilocybin administration, with significant response to long treatment maintained in 75% of participants and the remission rate was 58% at the 12-month follow-up. The drug also withstands comparison with classical antidepressant treatment, as double administration of psilocybin (separated by a 3-week interval) was as efficient as a 6-week chronic treatment with escitalopram [156,157].

Besides depression, numerous clinical studies have shown the therapeutic potential of psychedelics in treating disorders including addiction to alcohol [94,158] and nicotine [159,160,161], anorexia nervosa [162], distress and anxiety concerning death [163,164,165,166], obsessive–compulsive disorder [167], and post-traumatic stress disorder (PTSD) [168]. Psilocybin significantly increased abstinence and decreased craving up to 36 weeks after administration in alcohol-dependent participants [158] and elicited alterations in their relationship with alcohol [94]. Administration of moderate (20 mg/70 kg) and high (30 mg/70 kg) doses of psilocybin was a potentially efficacious adjunct to current smoking cessation treatment and promoted long-term smoking abstinence [159,160,161]. Classical psychedelics including psilocybin alleviated symptoms of anorexia nervosa that relate to serotonergic signaling and cognitive inflexibility [162]. Administration of low (1 or 3 mg/70 kg), moderate (0.2 mg/kg), or high (22 or 30 mg/kg) doses of psilocybin produced immediate, substantial, and sustained improvements in anxiety and depression in patients with life-threatening cancer at 1 to 6 months after treatment [163,164,165]. A similar reduction in anxiety was demonstrated in LSD-assisted psychotherapy [166]. Psilocybin in single doses ranging from sub-hallucinogenic (100 µg/kg), medium (200 µg/kg), and high (300 µg/kg) relieved symptoms of obsessive–compulsive disorder in a controlled clinical environment [167]. Classical psychedelics such as psilocybin and LSD showed significant potential for treating PTSD. However, their unpredictable psychological effects might not make them the best candidates compared with MDMA [168].

Serotonergic hallucinogens, acting on 5-HT2A receptors, induce perceptual and behavioral alterations possibly related to psychotic symptoms. It is suggested that the psychotomimetic properties of psychedelics are mediated by an alteration in thalamocortical activity, which is the neurobiological basis of schizophrenia [169,170]. Given the enhancing effect of psychedelics on neural plasticity and their effects on inflammatory processes, it is possible that psychedelics could have a role in treating cortical atrophy and cell loss in schizophrenia and the negative symptoms associated with this illness. It is thus possible that microdosing, in which psilocybin is taken in one-tenth of the typical psychedelic dose, has no psychosis-inducing effects while it may have positive effects on mood, may alleviate depression and anxiety, and improve cognitive function [171].

## 6. Conclusions and Future Questions

The presented data support the hypothesis about a greater risk associated with the use of Novel Psychoactive Substances (i.e., NBOMes) in comparison with naturally occurring psychedelics. Therefore, the potential efficacy of these substances for medical treatment is ambiguous or low in comparison with psilocybin. The reviewed studies allow for the characterization of the effects of selective 5-HT2A/C receptor agonists on excitatory neurotransmitter systems and establish how those changes may translate into behavior and damage inflicted upon the central nervous system.

A similar approach was then applied to a compound with a broader receptor profile (psilocybin), uncovering its subtler effects resulting from the interplay of 5-HT1A and 5-HT2A receptors, with possible beneficial influence (i.e., lasting anxiolytic effect).

Mechanistically, psychedelics induce changes in neuronal structure that activate BDNF/TrkB/mTOR signaling crucial to synapse formation by stimulating 5-HT2A receptors [122,123]. The localization of 5-HT2A receptors highly expressed on layer V pyramidal neurons of the PFC could explain why psychedelics do not promote plasticity in the mesolimbic pathway and are not addictive. However, it is important to consider the potential risks associated with excessive release of glutamate leading to excitotoxicity and cell atrophy. Another question that needs to be resolved is whether the subjective effects of psychedelics are a critical component of their therapeutic mechanism. The development of non-hallucinogenic compounds such as analogs of 5-MeO-DMT, 6-MeO-isoDMT or tabernanthalog (TBG), which exhibit reduced hallucinogenic potential while retaining psychoplastogenic potency, may greatly improve the accessibility of therapy with psychedelics [172]. The recent discovery of linking intracellular 5-HT2A receptors with the induction of plasticity by molecules having lipophilic properties can lead to new drug discovery [173].

## Figures and Tables

**Table 1:** Serotonin receptor interactions with some phenethylamine and indoleamine psychedelics (according to [20,21]).

Compound/Receptor Subtype	Ki ± SD (nM)			
	5-HT1A	5-HT2A	5-HT2B	5-HT2C
**Phenethylamines**				
Mescaline	4600 ± 400	6300 ± 1800	>2000	17,000 ± 2000
25I-NBOMe	1800 ± 300	0.6 ± 0.2	130 ± 80	4.6 ± 2
25B-NBOMe	3600 ± 300	0.5 ± 0	10 ± 10	6.2 ± 2.2
**Indoleamines**				
Psilocin	123 ± 20	49 ± 10	>20,000	94 ± 9
DMT	75 ± 20	237 ± 40	34 ± 32	424 ± 150
LSD	3 ± 0.5	4 ± 1	12,000 ± 400	15 ± 3

**Table 2:** Abbreviations and chemical names of some NBOMe compounds [35].

Abbreviation	Chemical Name
25B-NBOMe	2-(4-bromo-2,5-dimethoxyphenyl)-N-(2-methoxybenzyl)ethanamine
25C-NBOMe	2-(4-chloro-2,5-dimethoxyphenyl)-N-(2-methoxybenzyl)ethanamine
25D-NBOMe	2-(2,5-dimethoxy-4-methylphenyl)-N-(2-methoxybenzyl)ethanamine
25I-NBOMe	2-(4-iodo-2,5-dimethoxyphenyl)-N-(2-methoxybenzyl)ethanamine
25N-NBOMe	2-(2,5-dimethoxy-4-nitrophenyl)-N-(2-methoxybenzyl)ethanamine

## Data Availability

Data sharing is not applicable to this article. No new data were created or analyzed in this study.
